# On the Frontiers of Breast Cancer Diagnosis and Treatment: Current and Future Directions in a Rapidly Changing Field

**DOI:** 10.3390/medicina58081026

**Published:** 2022-07-31

**Authors:** Jimmy T. Efird, Charulata Jindal, Tithi Biswas

**Affiliations:** 1 VA Cooperative Studies Program Coordinating Center, Boston, MA 02130, USA; tithi.biswas@uhhospitals.org; 2 Department of Radiation Oncology, Case Western Reserve University School of Medicine, Cleveland, OH 44106, USA; 3 Harvard Medical School, Harvard University, Boston, MA 02115, USA; charujindal@gmail.com; 4 Department of Radiation Oncology, University Hospitals Cleveland Medical Center, Case Western Reserve University, Cleveland, OH 44106, USA

Breast cancer (BCa) represents a medically heterogeneous group of malignancies, with differing biological and genetic makeups [1,2]. This malignancy unequally affects women of different races, ethnicities, and economic backgrounds [3,4]. Because of its diverse prevalence and subtypes, BCa often poses diagnostic and treatment challenges. In previous generations, guidance tended to lack specificity and direction in the management of BCa [5]. 

The landscape of BCa today has moved well beyond its historic limitations, with the advent of encouraging innovations not only in precision diagnosis and treatment, but most importantly in understanding the biology of the disease. Novel developments in circulating tumor cells (CTCs) and circulating tumor DNA (ctDNA) technology (i.e., liquid biopsies) has led to a less-expensive and non-invasive means for predicting disease severity and survival potential in BCa patients [6,7,8]. The refinement of tumor profiling using artificial intelligence and machine learning tools has likewise enabled the prediction of drug response with selected miRNA isoforms [9]. 

Phenotype and genotype, with the aid of tissue microarray data, currently classifies BCa into distinct subgroups. This reflects the 8^th^ edition of the AJCC cancer staging manual for BCa where, in addition to classic anatomic Tumor, Node and Metastasis (TMN) categories, prognostic groups now incorporate information on grade (i.e., Nottingham histological score), receptor expression (i.e., ER, PR, and HER2 status), and results from a multigene panel assay [10]. With the adoption of these guidelines, BCa staging more accurately reflects the underlying biology and inherent tumor aggressiveness than previously possible. 

Inroads in pharmaceutical therapies for BCa have been equally impressive, with the well-tested and currently available inhibitors of CDK4/6 and AKT. Furthermore, recent regulatory approvals include: (1) TRODELVY (sacituzumab govitecan) for triple negative BCa (TNBC); (2) ENHERTU (trastuzumab deruxtecan) for unresectable or metastatic HER2-positive BCa; (3) TUKYSA (tucatinib) for advanced and metastatic HER2-positive BCa, including patients with brain metastases; and (4) PIQRAY (alpelisib) for HR^+^/HER2^−^ advanced or metastatic BCa with PIK3CA mutations who have progressed after initial aromatase inhibitors [11,12]. 

Cellular immune function also plays an important but intricate role in BCa. On the treatment side, both immune checkpoint inhibitors (targeting programmed death protein-1/programmed death protein-ligand 1) and tumor-infiltrating lymphocytes have demonstrated clinical benefit in BCa, especially TNBC disease [13,14]. As a potential predictive marker for BCa, cytotoxic T-lymphocyte-associated antigen 4 (CTLA-4/CD152), a member of the Immunoglobulin superfamily and negative regulator of T-cell activation, is at the forefront of targeted compounds receiving research attention. Specifically, in a meta-analysis based on 52 case-referent studies and stratified by ethnicity, the single nucleotide polymorphisms CTLA-4 +49 A/G has been associated with a statistically decreased risk of BCa [15]. 

Another exciting area of progress has been in polymeric nanotechnology [16,17]. Multiple drug resistance and the presentation of unwanted side effects, attributable to inadequate drug concentrations at the tumor site, has limited the effectiveness of various BCa drugs. Nanoscale-based pharmaceuticals, focusing on the tumor microenvironment and tumor disseminate cells, have shown promise for overcoming these limitations. For example, the combined incorporation of quercetin (a naturally occurring flavonoid manifesting antioxidant, anticarcinogenic, anti-inflammatory, antiallergic, and antiviral properties), scorpion venom peptides (known to induce apoptosis), and the proprietary compound Phospholipon 90H into a nano-based delivery system has displayed antiproliferative efficacy in a cell line derived from human BCa MCF-7 cells [18]. This optimized formula resulted in significant cell cycle arrest at the S phase and increased levels of caspase-9, Bax, Bcl-2, and p53 mRNA expression.

Therapeutic advances extend beyond the above-mentioned drugs involving local therapy. Intraoperative radiation therapy (IORT), delivering a single dose of radiation during breast conserving surgery (BCS), has become an accepted alternative to whole breast irradiation (WBI) among patient with early stage BCa [19,20]. Additionally, hypo- and ultra-fractionated radiation after surgery has now become standard of care, without decreasing local control or increasing long term late toxicities [21]. In fact, most modern trials have shown the risk of local failure remains extremely low with a breast conserving approach. There are several ongoing trials testing whether the best prognostic group of BCa patients can avoid adjuvant radiation altogether (NCT03878342) [22], NCT02889874 [23]). 

On the surgical frontline, autologous and implant-based breast reconstructions are innovations currently available for the rebuilding of the breast, typically on the same day as surgery [24]. Indeed, oncoplastic breast surgery has become a hallmark in the post-surgical management of BCa patients [25]. Additionally, sentinel lymph node biopsy has replaced morbid full axillary nodal dissection in most cases of early BCa, thereby reducing long-term complications, without decreasing local control. 

BCa continues to be the most commonly diagnosed malignant tumor among women worldwide [26]. In spite of advances in the field, the death rate remains high for advanced and metastatic disease. Many questions persist regarding the diagnosis and treatment of this cancer, especially given its intricate molecular subtypes and varying pathologies [27]. In the search for solutions, we expect that research will progress forward with the discovery and implementation of novel targeted compounds and other breakthrough therapies. Early BCa diagnosis will also benefit from research involving innovative imaging techniques and efforts to reduce the cost of (and correspondingly, increase the access to) these methods. The ongoing transition from traditional localized therapy to systemic-based approaches, based on a patient’s molecular and genetic profile, holds promise for future advances that ideally will improve patient survival and quality of life [28].
